# Identification and verification of hub biomarkers and immune-related pathways participating in the trabecular meshwork after using corticosteroid

**DOI:** 10.1371/journal.pone.0331281

**Published:** 2025-09-25

**Authors:** Liwen Wang, Di Wu, Ziwei Ma, Di Song, Yuanqiang Sun, Xuefeng Pan, Junhui Zhang

**Affiliations:** 1 Department of Ophthalmology, Huzhou Central Hospital, Huzhou, China; 2 The First People’s Hospital of Huzhou, The First Affiliated Hospital of Huzhou Teacher College, Huzhou, China; Oregon Health and Science University, UNITED STATES OF AMERICA

## Abstract

**Background:**

Clinically, ocular application of corticosteroids is common.But it always result in higher Intraocular pressure (IOP).The potential molecular events are not clearly explained.It was reported that immune cells may cause higher IOP.This study aimed to identify the central gene and immune-related pathways in ocular tissue treated with corticosteroids.

**Methods:**

The GSE124114 and GSE37474 was obtained from Gene Expression Omnibus database. Hub gene were acquired by differential expression analysis (DEGs).Weighted gene co-expression network analysis (WGCNA) identified hub gene modules associated with elevated intraocular pressure (IOP).CIBERSORT was utilized to evaluate the presence of immune cells in ocular tissue.R (version 3.6.1) was used to carry out enrichment analysis for DEGs.The STRING database constructed the protein-protein interaction network for differentially expressed genes (DEGs).The expression of hub genes was validated using the combined datasets GSE6298 and GSE65240. ROC analysis was conducted for clinical diagnosis in patients with elevated IOP.

**Results:**

30 DEGs were recognized. Gene ontology (GO) and Kyoto Encyclopedia of Genes and Genomes (KEGG) pathway analyses revealed that these DEGs were mainly associated with the positive regulation of cytokine production and pathways related to phenylalanine metabolism.Two hub modules were enriched in the rheumatoid arthritis and AGE-RAGE signaling pathways associated with diabetic complications. Analysis of the protein-protein interaction (PPI) network identified TSC22D3 and FKBP5 as related hub genes. Analysis of immune infiltration indicated a significant association between TSC22D3 and Macrophages M0 (R = 0.75, p = 0.018).The result of ROC for FKBP5 was 0.913.Consolidated GSE6298, GSE65240 database verified that FKBP5 gene was associated with IOP.In the results of qPCR, relative mRNA level of FKBP5 were incread.

**Conclusions:**

Two key genes (TSC22D3, FKBP5) are involved in the molecular mechanisms in corticosteroids-induce higher IOP. TSC22D3 was probably associated with the immune response in ocular tissue. FKBP5 may be a potential novel diagnostic gene for higher IOP.

## Introduction

Glaucoma, a primary cause of irreversible blindness globally, impacted around 76 million individuals in 2020, with projections exceeding 111 million by 2040 [1]. Ocular hypertension serves as an independent risk factor for the onset and progression of glaucoma.Ocular hypertension is categorized into primary and secondary types. Primary ocular hypertension usually happens due to the special anatomy, while there are many reasons for secondary ocular hypertension, including ocular inflammation and trauma, pigment dispersion and exfoliation, neovascularization, dense cataract formation, corneal pathologies and the use of corticosteroids [2]. Ocular inflammation is a primary contributor to secondary elevated intraocular pressure (IOP). Topical glucocorticoids are widely used for their immunosuppressive and anti-inflammatory properties. Studies indicate that approximately one-third of the general population experiences increased intraocular pressure (IOP) after using topical corticosteroids for two weeks or longer [3]. This elevation is attributed to corticosteroids potentially reducing outflow, as corticosteroid receptors have been identified in the trabecular meshwork [4,5]. Consequently, the association between corticosteroids and elevated IOP is widely recognized.however, the specific mechanism remains unclear.

Corticosteroids are frequently utilized in clinical ophthalmology. Uveitis, an immune-related condition, necessitates prolonged corticosteroid therapy. Prolonged steroid use often leads to secondary glaucoma due to increased intraocular pressure (IOP) [6]. However, a limitation existed in the monitoring mode during prolonged use of corticosteroid-related eye drops until an increase in intraocular pressure (IOP) occurred. Discontinuing hormone use may help lower IOP, but there is a risk of disease recurrence. Therefore, balancing corticosteroid use with complication prevention is crucial. Investigating the molecular mechanisms underlying corticosteroid-induced ocular hypertension is essential for developing effective diagnostic methods and identifying reliable molecular markers to prevent increased intraocular pressure during hormone therapy. Given that glucocorticoids primarily influence target gene expression via glucocorticoid receptors, investigating their interaction with glucocorticoid application, gene expression alterations, and secondary ocular hypertension is crucial. Gene expression microarrays are widely utilized to study gene expression profiles, offering a novel method for investigating genes with diverse applications in drug-based molecular targeting. The Gene Expression Omnibus (GEO) [7] recently released a substantial amount of data. Combining these datasets enables researchers to gain deeper insights into molecular mechanisms.

The advancement of next-generation sequencing technology has led to the development of various computational strategies for identifying disease-specific biomarkers. Weighted gene co-expression network analysis (WGCNA) is an innovative systems biology approach that enables the construction of scale-free gene co-expression networks and the identification of gene modules and hub genes [8]. Analyzing the relationship between modules and clinical factors allows us to identify modules linked to disease phenotypes [9].

This study utilized four microarray datasets (GSE124114, GSE37474, GSE6298, GSE65240) from the GEO database, comprising 41 samples: 16 healthy controls and 25 trabecular meshwork samples co-incubated with corticosteroids.Datasets GSE124114 and GSE37474 were combined to identify differentially expressed genes (DEGs) based on the most significant log2|FC| values, using R software (version 3.6.1) packages for analysis. Furthermore, the enriched pathways of DEGs were analyzed using the gene ontology (GO) and Kyoto Encyclopedia of Genes and Genomes (KEGG) databases. Hub gene modules linked to elevated IOP were identified using weighted gene co-expression network analysis (WGCNA). The primary module exhibiting the strongest correlation with corticosteroid induction was identified. Within this module, genes meeting the stringent criteria of |gene significance (GS)| > 0.2 and |module membership (MM)| > 0.8 were classified as hub genes. Subsequently, DEGs were integrated with hub gene modules to identify the central hub gene. Subsequently, the STRING online database was utilized to analyze the predicted associations within a specific set of proteins using the protein-protein interaction (PPI) network. GSE37474, being the sole database for trabecular meshwork and corneoscleral tissue, was selected for immune infiltration analysis using the CIBERSORT algorithm following data batch correction and standardization. The datasets GSE6298 and GSE65240 were combined and underwent batch correction to validate key genes. The hub markers’ accuracy in monitoring corticosteroid-induced effects was evaluated using the receiver operating characteristic (ROC) curve. This study initially investigates the mechanism behind corticosteroid-induced ocular hypertension, offering insights into the pathological processes, diagnostic approaches, and treatment strategies for glucocorticoid-induced glaucoma.

## Materials and methods

### Search strategy

**Fig 1:**
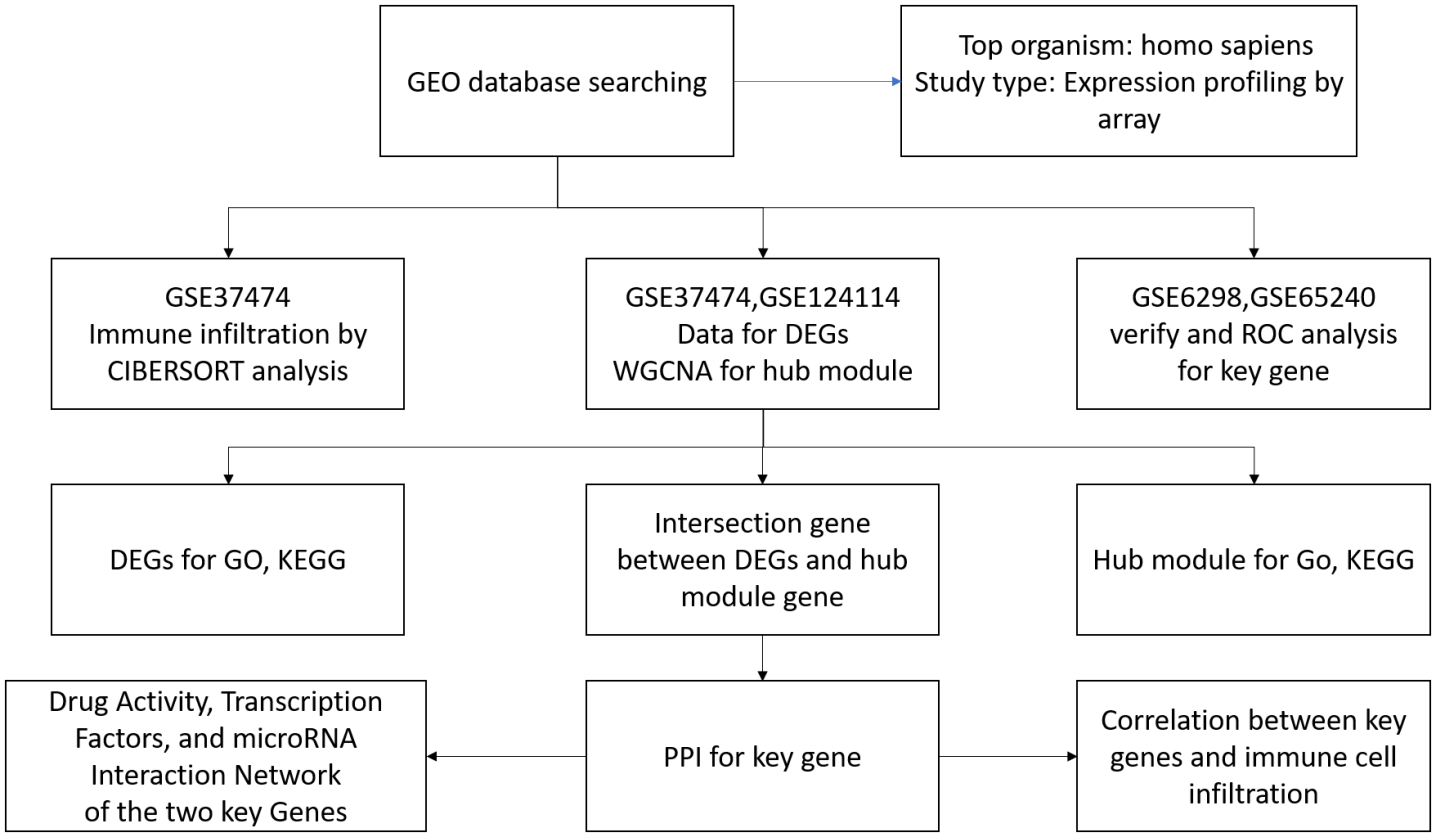
Serials selection process.

A search of the GEO database (https://www.ncbi.nlm.nih.gov/geo/), using the keywords “corticosteroids” and “glaucoma” yielded four results. By limiting the entry type to series, the study type to expression profiling by array, and the tissue sources to Homo sapiens, four unrelated items were excluded from the study. Subsequently, gene expression profiles from four series—GSE37474, GSE6298, GSE65240, and GSE124114—were obtained from the platforms GPL6244, GPL17077, GPL570, and GPL4564. Fig 1 showed the details of the selection process.

### Microarray data information

The platform for GSE37474 was GPL570 [HG-U133_Plus_2] Affymetrix Human Genome U133 Plus 2.0 Array, which included 5 tissues (trabecular meshwork and corneoscleral) from Corticosteroids-induced sample and other 5 from health sample.The platform for GSE124114 was GPL6244 [HuGene-1_0-st] Affymetrix Human Gene 1.0 ST Array [transcript (gene) version], which included 9 trabecular meshwork cell samples induced by Corticosteroids and other 9 from the control sample.The GSE65240 dataset utilized the GPL17077 Agilent-039494 SurePrint G3 Human GE v2 8x60K Microarray platform, comprising three trabecular meshwork cell samples treated with corticosteroids and three control samples.GSE6298 utilized the GPL4564 Stanford Human cDNA SHEW platform, comprising 9 trabecular meshwork cell samples. The correction results were presented in supplementary materials. TXT files were created by downloading the platform and series matrix files from GEO.The downloaded files were processed using R software (version 3.6.1).

### Integration of microarray data and DEGs

The analysis utilized raw datasets from GSE124114 and GSE37474. The microarray datasets were batch normalized using R software (https://bioconductor.org/biocLite). R was used to save the analysis results as a TXT file via the limma package (biocLite ‘limma’).The DEGs in control and experimental samples were analyzed using the limma package in R software.The instruction code is run using R programme.Genes that were upregulated or downregulated were collected separately and used for further investigation.The downloaded data, encompassing both the platform and matrix series, was processed using the R program.

### Data processing and identification of DEGs

Using the same limma programme, a volcano plot was constructed to depict all the up-regulated and down-regulated DEGs. Hierarchical clustering was performed using the heatmap program, restricting the analysis to 30 differentially expressed genes (DEGs). The findings were visualized using a heatmap.The probe name ID was converted to a gene symbol using Perl (v5.30.0) and a genome-wide annotation library from Bioconductor.Hs.e.g.,db.html) and subsequently saved as a TXT file. Differentially expressed genes (DEGs) were identified based on adjusted P-values of 0.05 and a log fold change (logFC) greater than 1.5.

### GO and KEGG pathway enrichment analyses of DEGs

The clusterProfiler package [10] was employed for GO and KEGG pathway enrichment analysis on the DEG, using a Bonferroni-corrected p-value (Q) < 0.05 as the significance threshold to assess the biological functions and pathways influenced by differential gene sets in corticosteroid-induced samples. GO keywords with p < 0.05 were deemed statistically significant, encompassing biological processes (BPs), cellular components (CCs), molecular functions (MFs), and KEGG pathways.The GO and KEGG analysis results were visualized using the “ggplot2” R package.

### Construction of co-expression modules by WGCNA

Gene co-expression networks were constructed using the aggregated expression profile datasets GSE124114 and GSE37474. We conducted GO and KEGG pathway enrichment analyses on the target modules to elucidate the biological significance of the Corticosteroids-related module. Hub gene sets with a gene importance greater than 0.2 and module membership exceeding 0.8 were analyzed. The hub gene sets were intersected with DEGs for further analysis.

### GO enrichment and KEGG pathway analysis of genes in significant module

To characterize the functional attributes of genes within each co-expression module, we performed GO enrichment analysis and KEGG pathway analysis. These analyses systematically identified significantly enriched biological processes, cellular components, and molecular functions. A hypergeometric test was used to evaluate statistical significance, with an adjusted p-value (q-value) that was less than 0.05. Top 5 GO enrichment findings were visualized using bar graphs, and both GO and KEGG pathway analyses were conducted with the “ggplot2” R package.

### PPI network and the identification of intersection genes between hub gene and DEGs

STRING (https://string-db.org/cgi/input.pl) is an online database encompassing over 24.6 million proteins and 3.1 billion interactions across 5090 organisms [11]. We utilized STRING (version 11.0) to construct PPI networks for the intersection of hub genes and DEGs.The interaction score threshold was established at a medium confidence level of 0.700.

### Analysis of key gene expression and their receiver operating characteristic (ROC) curves

Receiver operating characteristic (ROC) curves were generated using R to evaluate the ability of key genes to differentiate between corticosteroid-induced trabecular meshwork and control samples.The expression levels and diagnostic significance of the hub genes were validated using an external dataset, which combined GSE6298 and GSE65240.

### CIBERSORT analysis of immune infiltration

Following data batch correction and standardization, we selected GSE37474 for immune infiltration analysis using the CIBERSORT method, as it was the only database available for trabecular meshwork and corneoscleral tissue.The CIBERSORT12 software was utilized to obtain the immune cell infiltration matrix [12]. This study examined twenty-two types of immune cells, including macrophages M2, plasma cells, neutrophils, activated mast cells, CD8 T cells, macrophages M1, gamma delta T cells, memory B cells, monocytes, naive B cells, follicular helper T cells, activated NK cells, resting dendritic cells, activated CD4 memory T cells, naive CD4 T cells, resting NK cells, regulatory T cells (Tregs), activated dendritic cells, eosinophils, macrophages M0, resting CD4 memory T cells, and resting mast cells.

### Real-time PCR analysis

Human Trabecular Meshwork (TM) cells used in this study were sourced from Wenzhou Gaodian Biotechnology Co., LTD. Four batches were cultured for 14 days, with one group receiving 500 nM DEX treatment and the other serving as a control. mRNA levels were assessed via qPCR (7300 Real Time PCR System, Applied Biosciences) utilizing NCBI-designed specific primers for FKBP5 and TSC22D3. The Actin gene was employed as a reference gene. The data were standardized relative to untreated cells on day 0. Table 1 lists the primers utilized in the qPCR reactions.

**Table 1:** Primers used for qPCR.

Type	Sequence
FKBP5 F	CGTCCCAGAGGGGGAA
FKBP5 R	GATTGTCGCTTCGTAGTCCC
TSC22D3 F	CTTCAGAGCCGGTGCCT
TSC22D3 R	GGATCTCCACCTCCTCTCTC

### Examination of transcription factor and microRNA interactions

To analyze target gene transcription factors, we built a network between transcription factors and genes using the miRTarBase [13]. ChEA(https://dev.networkanalyst.ca/NetworkAnalyst/Secure/AnalysisOverview.xhtml) was utilized to analyze the targets of validated microRNA (miRNA)-mRNA interactions.

## Results

**Fig 2:**
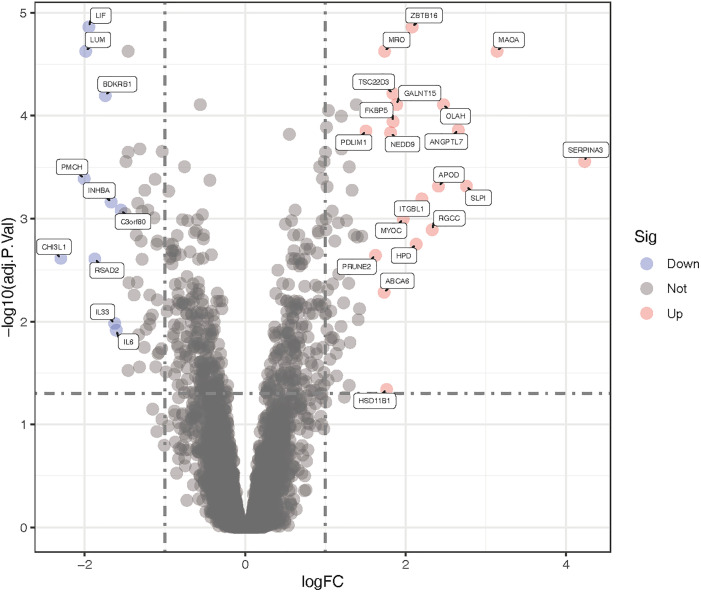
Volcano plot of the differentially expressed genes between Corticosteroids-induced and normal sample. Black points indicate an adjusted P > 0.05. Blue points indicate down-regulated genes with an adjusted P < 0.05. Red points indicate up-regulated genes with an adjusted P < 0.05.

**Fig 3:**
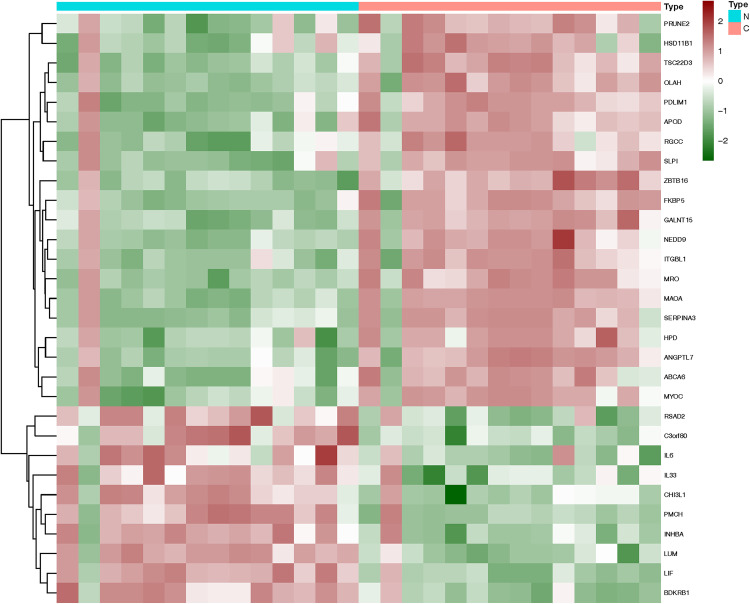
Heatmap displaying the top 30 differentially expressed genes (DEGs) based on adjusted P-value and log fold change (logFC). Red signifies elevated gene expression, whereas green denotes reduced gene expression. N control group; C Corticosteroids group.

Compared with the control, an aggregate of 30 DEGs was acquired, of which 20 were up-regulated and 10 were down-regulated respectively (Fig 2). Table 2 separately presents the upregulated and downregulated differentially expressed genes (DEGs). The purple spot indicates genes that are downregulated, whereas the red ones signify upregulated genes. A heatmap illustrating the 20 up-regulated and 10 down-regulated DEGs was generated using R-heatmap software, as depicted in Fig 3.

**Table 2:** Differentially expressed genes (DEGs) exhibiting both increased and decreased expression levels.

DEGs	Gene symbol
Down-regulated	CHI3L1, PMCH, LUM, LIF, RSAD2, BDKRB1, INHBA, IL33, IL6, C3orf80
Up-regulated	PDLIM1, PRUNE2, ABCA6, MRO, HSD11B1. NEDD9, TSC22D3, FKBP5, GALNT15, ZBTB16, HPD, ITGBL1, RGCC, APOD, OLAH, ANGPTL7, SLPI, MAOA, SERPINA3, MYOC

DEGs: Differentially expressed genes.

### GO term and KEGG pathway enrichment analysis of DEGs

**Fig 4:**
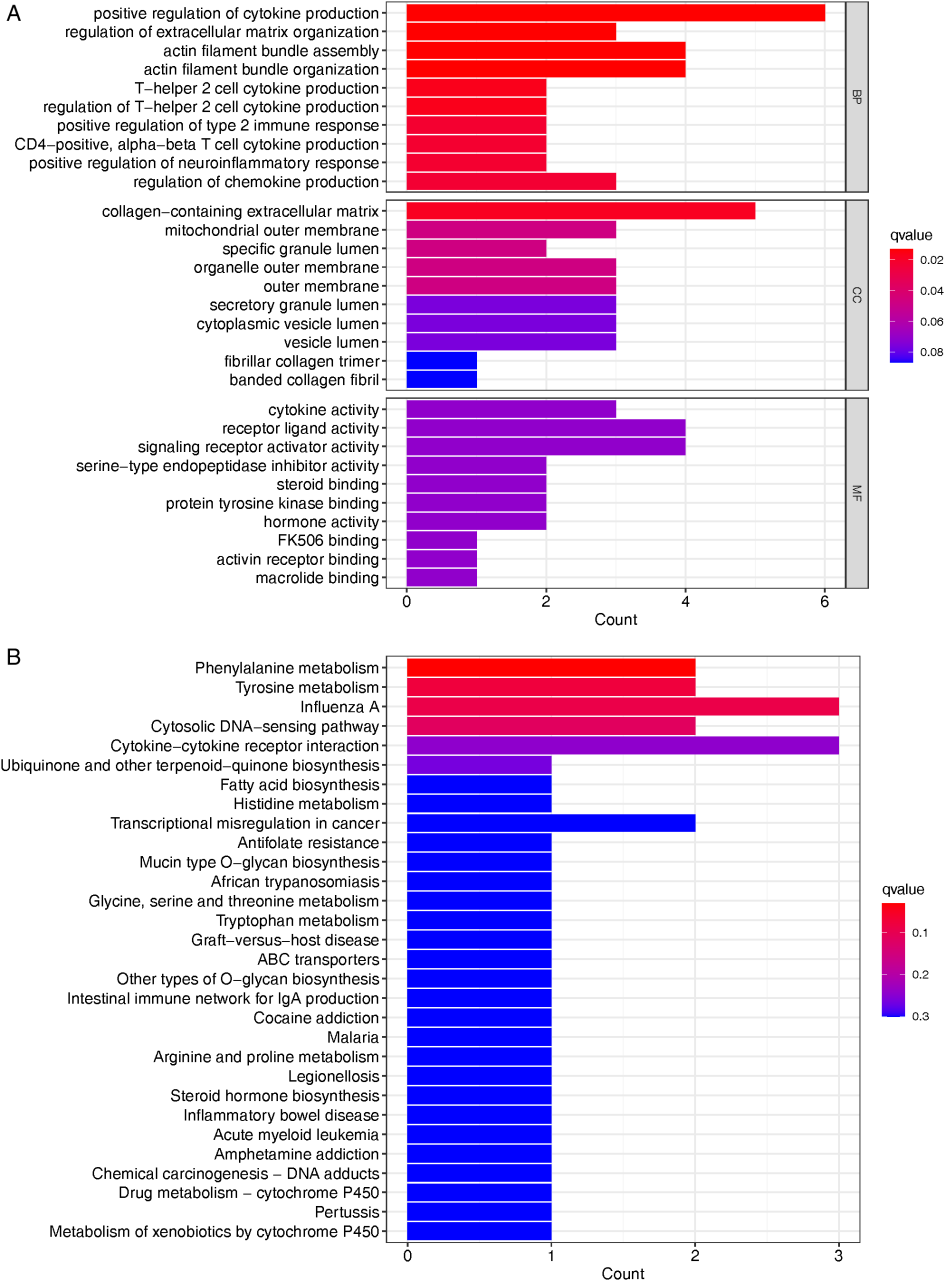
Analysis of DEGs using GO enrichment and KEGG enricment. A. Analysis of DEGs using GO enrichment. B. KEGG pathway enrichment analysis of DEGs^‌^.

The GO term and KEGG pathway enrichment analysis of up and down-regulated genes with an adjusted P value of < 0.05 were obtained respectively. Table 3 and Fig 4A display the GO term results for the corticosteroids group. Table 3 and Fig 4B display the KEGG enrichment analysis results of DEGs in the corticosteroids group. The DEGs were primarily associated with the enhancement of cytokine production, modulation of extracellular matrix organization, assembly and organization of actin filament bundles, T-helper 2 cell cytokine production, and phenylalanine metabolism. KEGG analysis revealed that the DEGs were exclusively enriched in the chemokine signaling pathway.

**Table 3:** TOP 5 GO gene ontology and KEGG, DEGs differentially expressed genes.

ID	Description	Adjusted P-values	Gene symbol	Count
**GO:0001819**	**positive regulation of cytokine production**	**4.42E-05**	**LUM, RGCC, CHI3L1, RSAD2, IL33, IL6**	**6**
**GO:1903053**	**regulation of extracellular matrix organization**	**4.43E-05**	**ANGPTL7, RGCC, IL6**	**3**
**GO:0051017**	**actin filament bundle assembly**	**7.19E-05**	**PDLIM1, NEDD9, MYOC, RGCC**	**4**
**GO:0061572**	**actin filament bundle organization**	**0.019687**	**PDLIM1, NEDD9, MYOC, RGCC**	**4**
**GO:0035745**	**T-helper 2 cell cytokine production**	**0.000155**	**RSAD2, IL6**	**2**
**hsa00360**	**Phenylalanine metabolism**	**0.03144**	**MAOA/HPD**	**2**

### WGCNA for identification of hub genes and PPI for key genes

**Fig 5:**
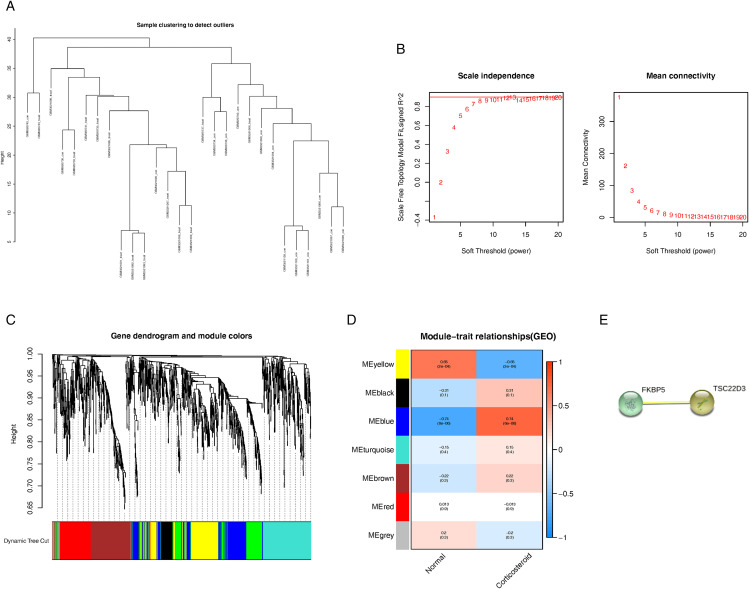
WGCNA Co-expression Network Analysis Steps and Module Identification as well as the results of PPI. A. Dendrogram of sample clustering for GSE124114 and GSE37474 to identify outliers. B. Evaluation of the scale-free fit index on the left and the mean connectivity on the right for determining various soft thresholding powers. C. The clustering dendrogram illustrates gene grouping in corticosteroid-induced traits, with each color indicating a distinct co-expression gene module. D. The heatmap illustrates the relationship between the module and traits caused by corticosteroids. E. Intersection genes revealed by PPI.

**Fig 6:**
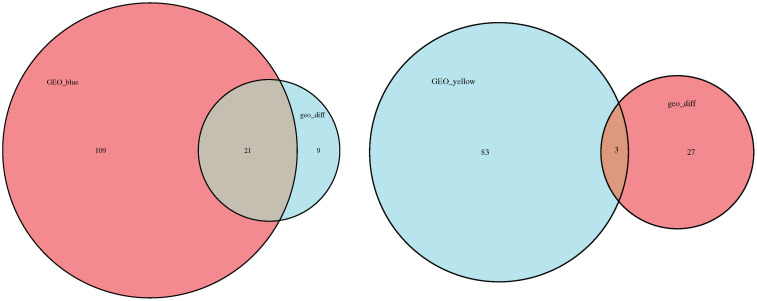
Venngene between the blue module and DEGs(left). Venngene analysis between the yellow module and DEGs(right).

The sample clustering dendrogram of consolidated GSE124114 and GSE37474 was showed in Fig 5A, there was no significant difference observed among the samples included in the WGCNA. We identified 8 as the most suitable soft threshold power by considering the scale-free topology model and mean connectivity (Fig 5B). Fig 5C displays the dendrogram of the gene cluster, with each leaf symbolizing a gene and each branch representing a co-expression module.The heat map (Fig 5D) demonstrates the correlation between various modules and traits induced by corticosteroids. We identified two modules, with the yellow and blue consensus module showing the strongest association with corticosteroids (correlation values of 0.65 and −0.74, respectively; p < 0.05). A total of 86 hub genes were discovered in the yellow co-expression modules, with gene significance higher than 0.2 and module membership greater than 0.8. Three genes overlapped with the DEGs. A total of 130 hub genes were identified in blue co-expression modules, characterized by a gene significance > 0.2 and module membership > 0.8. A total of 21 genes were found to overlap with the DEGs (Fig 6). The intersection genes between the hub module and DEGs were identified as INHBA, RGCC, and CHI3L1 within the yellow module, and ABCA6, ANGPTL7, APOD, FKBP5, GALNT15, HSD11B1, ITGBL1, LIF, LUM, MAOA, MRO, MYOC, NEDD9, OLAH, PDLIM1, PMCH, PRUNE2, SERPINA3, SLPI, TSC22D3, and ZBTB16. The interaction score threshold was established at a medium confidence level of 0.700. Following the creation of PPI networks for intersection genes, TSC22D3 and FKBP5 emerged as the most crucial genes(Fig 5E).

### Top 5 GO term and KEGG pathway enrichment analysis of yellow and blue module

**Fig 7:**
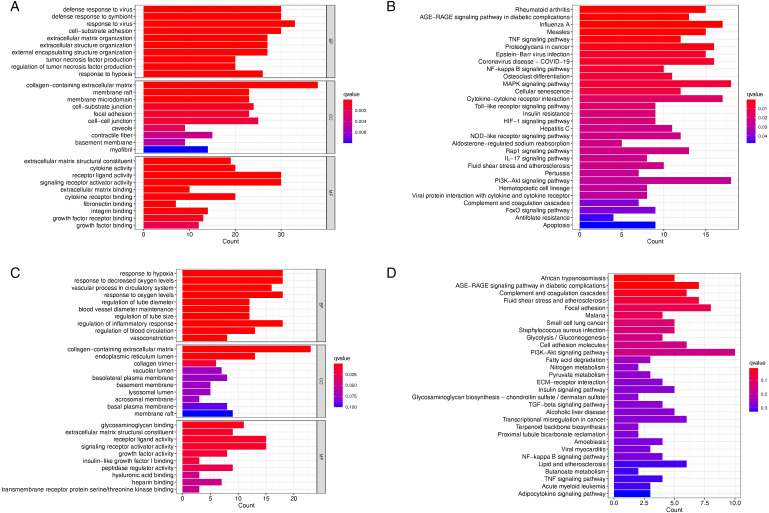
GO and KEGG enrichment analysis for the blue and yellow module. A. GO enrichment analysis for the blue module. B. KEGG enrichment analysis for the blue module. C. GO enrichment analysis for the yellow module. D. KEGG enrichment analysis of yellow module.

Fig 7A and C illustrates the GO term results for the yellow and blue modules. The visual analysis results of the KEGG enrichment of the yellow and blue modules were shown in Fig 7B and D. We applied the hypergeometric test and identified pathways as significantly enriched if they had p-values and corresponding Q-values both below 0.05. The yellow module genes were primarily associated with hypoxia response, decreased oxygen levels, vascular processes in the circulatory system, oxygen level response, and tube diameter regulation. KEGG analysis revealed that genes in the yellow module were enriched in pathways related to African trypanosomiasis, AGE-RAGE signaling in diabetic complications, and complement and coagulation cascades. The blue module genes were primarily associated with defense responses to viruses and symbionts, as well as cell-substrate adhesion and extracellular matrix organization. KEGG analysis revealed that genes in the blue module were significantly associated with pathways related to Rheumatoid arthritis, AGE-RAGE signaling in diabetic complications, Influenza A, Measles, and TNF signaling.

### Validation of expression levels of hub genes in consolidated GSE6298 and GSE65240

**Fig 8:**
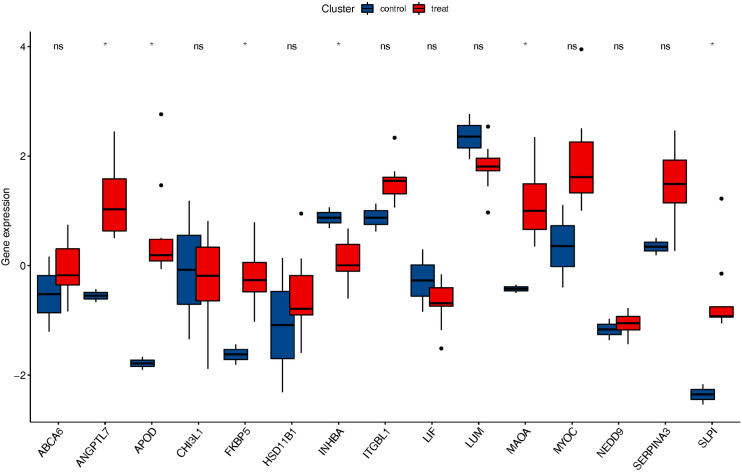
Validation of expression levels of Two hub genes in related GEO datasets. *: P < 0.05.

The key hub genes identified for corticosteroid-induced status were FKBP5 and TSC22D3, which were chosen for further analysis. Analysis of the combined GSE6298 and GSE65240 datasets revealed significantly elevated FKBP5 gene expression in the corticosteroids-induced group compared to the normal group (p < 0.05, Fig 8). However, TSC22D3 was not expressed in these datasets. Among the 24 intersection genes identified through WGCNA, ANGPTL7, APOD, MAOA, and SLPI exhibited significantly elevated expression in the corticosteroid-induced group compared to the normal group (p < 0.05 Fig 8). INHBA levels were notably reduced in the corticosteroid-induced group compared to the normal group (p < 0.05, Fig 8).

### ROC analysis of the selected two hub genes

**Fig 9:**
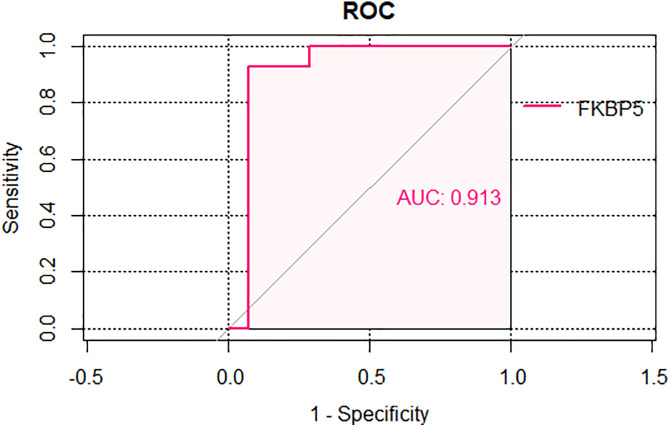
The ROC curve for the five hub genes in the combined datasets GSE124114 and GSE37474.

Next, we selected FKBP5 to do ROC analysis. The ROC curve was generated using the expression data from the combined GSE124114 and GSE37474 datasets to evaluate the genes’ monitoring value for corticosteroids (Fig 9). Fig 9 illustrates the ROC validation analysis results for the FKBP5 gene, with an AUC of 91.30%.

### Investigation of immune infiltration and the link of key genes with immune cells

**Fig 10:**
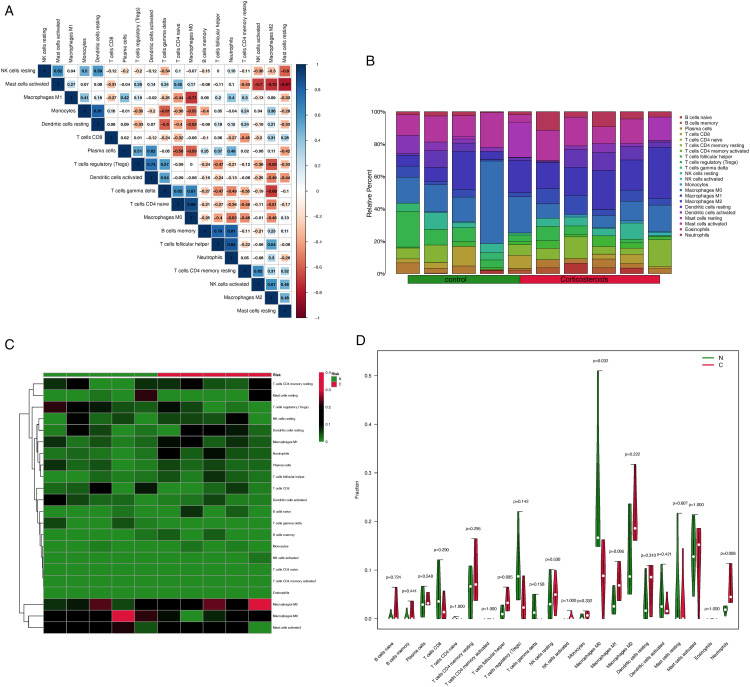
Results of CIBERSORT analysis of Gene Expression Omnibus database. A. Correlation matrix of immune cell infiltration levels in corticosteroid samples.Red signifies trends aligned with a negative correlation, while blue denotes trends aligned with a positive correlation between two immune cells. A larger dataset size indicates a stronger positive or negative correlation. B. The landscape of immune cell infiltration. C. The distribution of 22 immune cells in 10 gene matrix.Red indicates higher immune infiltration expression and green indicates lower expression. D. violin plot illustrating immune cell proportions across two groups. The green fusiform fractions on the left denote the normal group, while the red fusiform fractions on the right indicate the corticosteroid samples.

**Fig 11:**
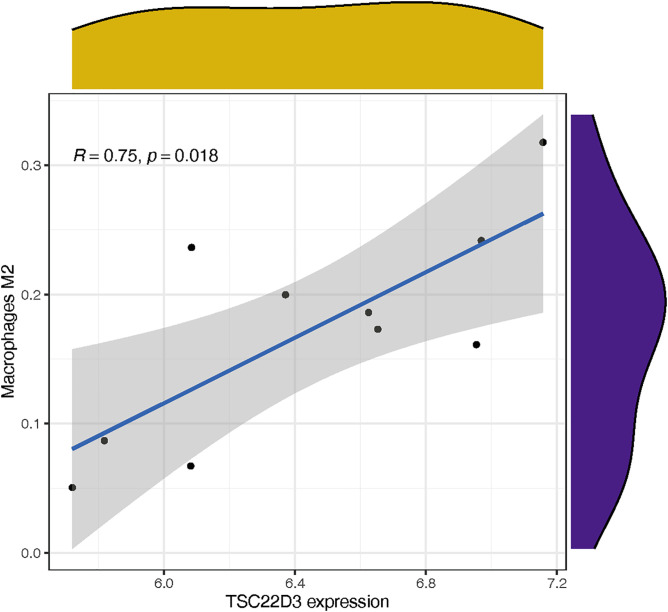
The association between TSC22D3 and M2 macrophages.

The heatmap (Fig 10A) indicated the presence of Macrophages M0 and Macrophages M1 exhibited a negative correlation with a value of −0.71. Macrophages M0 and Macrophages M2 exhibited a negative correlation with a value of −0.46. Macrophages M0 and naive CD4 T cells exhibited a negative correlation with a coefficient of 0.89. Following data batch correction and standardization of GSE37474, the correlation heatmap (Fig 10B) presented the findings from the analysis of 10 gene expression matrices, displaying the relative percentages of 22 immune cell types in Fig 10C. The violin plot analysis (Fig 10D) revealed a statistically significant reduction in Macrophages M0 infiltration in corticosteroid-induced samples compared to normal tissue (P = 0.032). TSC22D3 exhibited a positive correlation with Macrophages M2 (R = 0.75, p = 0.018) Fig 11).

### Development of a regulatory network for transcription factors and miRNAs associated with the two central hub genes

**Fig 12:**
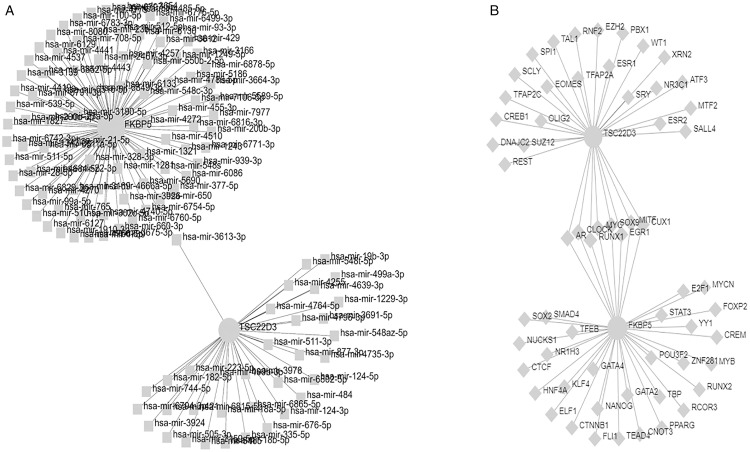
Illustrates the development of interaction network maps involving transcription factors (A) and miRNAs (B) for the hub genes TSC22D3 and FKBP5. A. illustrates the interaction network of two hub genes and their associated transcription factors. Circular nodes denote the key genes, while quadrate nodes indicate the transcription factors linked to these hub genes. B. A network diagram illustrating interactions between key genes and miRNAs, with circular nodes indicating key genes and diamond-shaped nodes representing miRNAs linked to the two central hub genes.

The network between transcription factors and genes using the miRTarBase [13] showed in Fig 12A. Fig 12B illustrates the miRNA-Gene interaction network based on ChEA data.

### qPCR analysis

**Fig 13:**
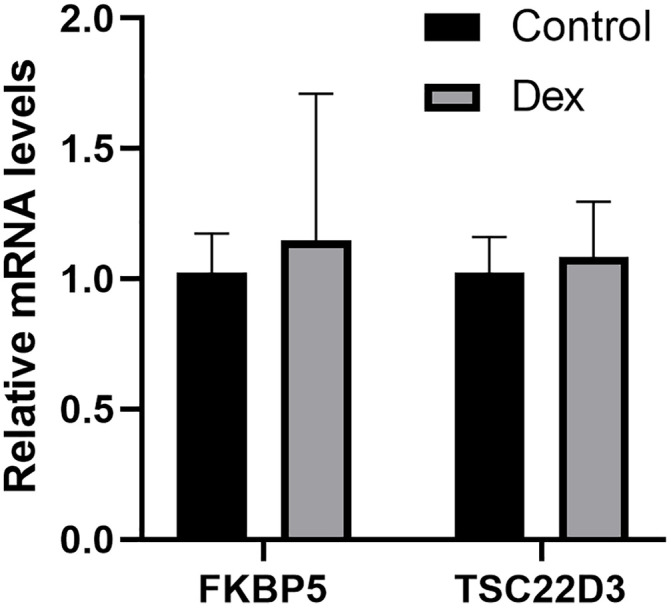
Quantification of FKBP5 and TSC22D3 mRNA expression levels with or without 500 nM DEX treatment for 14 days using real-time PCR.

Fig 13 demonstrates that qPCR analyses revealed an increase in mRNA levels of FKBP5 and TSC22D3 in Human Trabecular Meshwork Cells (HTMC), although these changes were not statistically significant compared to the control group.

## Discussion

Increased intraocular pressure (IOP) is an independent risk factor for the development and progression of glaucoma [14], a major cause of irreversible blindness worldwide. Elevated intraocular pressure (IOP) is linked to the use of corticosteroids, whether applied topically or systemically [15]. Corticosteroids are commonly used in treating eye illnesses, often resulting in ocular hypertension. Understanding the mechanism behind corticosteroid-induced ocular hypertension is crucial for managing corticosteroid-related ocular hypertension, as it remains unclear. The presence of corticosteroid receptors in the trabecular meshwork suggests that corticosteroids might reduce normal aqueous humor outflow [4]. The exact chemical mechanism by which corticosteroids affect aqueous fluid outflow and lead to increased intraocular pressure (IOP) remains unidentified. Previous studies have reported alterations in gene expression and structure following corticosteroid treatment. Selective laser trabeculoplasty (SLT) has been shown to effectively reduce corticosteroid-induced intraocular pressure (IOP) spike [5]. A biologic theory suggests that SLT treatment induces trabecular and endothelial cells to release chemotactic and vasoactive agents. These cytokines promote macrophage accumulation. Macrophages may impact on matrix metalloproteinase pathway, resulting in phagocytosis and extracellular matrix remodeling, which results in lower IOP [6]. Therefore, it is plausible that the immune system could affect aqueous flow. We sought to examine the possible molecular changes caused by corticosteroids in the immune system of the trabecular meshwork, and screening hub gene for early warning of IOP increasing or exploring a potential way to lower the increased IOP.

This study identified 30 differentially expressed genes (DEGs) potentially associated with increased intraocular pressure (IOP) following corticosteroid use. The enriched GO biological process terms for these genes predominantly include positive regulation of cytokine production [16,17], regulation of extracellular matrix organization [18], actin filament bundle assembly, actin filament bundle organization [19], and T-helper 2 cell cytokine production [18]. KEGG pathway analysis revealed a significant enrichment in Phenylalanine metabolism [20]. Using the WGCNA algorithm, genes linked to corticosteroid-induced changes in trabecular meshwork, which affect aqueous humor outflow and contribute to ocular hypertension, were categorized into two distinct modules: yellow and blue. The yellow module gene was primarily associated with various hypoxia responses. It was reported that hypoxia stimulated a robust elevation in vasorin (a kind of glycoprotein) expression, and vasorin suppressed TNF-α-induced cell death in trabecular meshwork cells [21]. The study indicates that corticosteroids may activate a response to hypoxia and affect trabecular meshwork cell function, thereby maintaining aqueous humor flow [5]. KEGG analysis revealed that these genes were predominantly enriched in the AGE-RAGE signaling pathway associated with diabetic complications. A study indicated that the AGE-RAGE signaling pathway could be the primary mechanism involved in reactive oxygen species-induced damage in TM cells related to diabetic complications [22]. The blue module gene analysis revealed a strong association with immune system processes, including defense responses to viruses and symbionts, cell-substrate adhesion [23], and inflammatory cytokines, which have been found to be elevated in the aqueous humor of glaucoma patients according to several studies [24–27]. Additionally, the blue module gene is associated with extracellular matrix organization. Zhou’s study indicates that glucocorticoids influence extracellular matrices in the trabecular meshwork, highlighting their significant role in glaucoma development [28]. The KEGG analysis revealed that the genes in the blue module were enriched in the AGE-RAGE signaling pathway associated with diabetic complications [22].

Further gene analysis is conducted based on these criteria: a) belongs in differential expression genes; b) gene significance exceeding 0.2 and module membership surpassing 0.8 in WGCNA; c) co-occurrence in the PPI network (middle confidence value = 0.700). The corticosteroid sample analysis identified TSC22D3 and FKBP5 as the key hub genes. Analysis of gene expression in the combined datasets GSE6298 and GSE65240 revealed a significant up-regulation of FKBP5. Subsequently, we conducted ROC curve analysis to assess the monitoring significance of FKBP5. The results demonstrated that these genes might serve as monitoring markers for the corticosteroids sample, because the AUC of these five genes was > 0.9 in the consolidated GSE124114 and GSE37474.

We identified transcription factors and regulatory microRNAs linked to the two burn diagnostic markers, offering novel targets for corticosteroid-induced treatment of elevated IOP.

FKBP5 is a 51-kDa immunophilin from the FK506-binding protein family, named for its capacity to bind the immunosuppressant FK506 [29]. Zhang et al. [30] reported that FKBP5 was involved in nuclear transport of the dominant-negative receptor for glucocorticoid (GRb), suggesting protective effects of FKBP5 against glucocorticoid-induced glaucoma. Variability in DNA demethylation levels of the FKBP5 gene following corticosteroid stimulation may influence the efficacy of negative feedback against glucocorticoid-induced signals [31]. Ewald et al. [32] identified a correlation between FKBP5 DNA methylation in peripheral blood and the brain in response to glucocorticoid exposure, suggesting that peripheral blood FKBP5 methylation could serve as a biomarker for glucocorticoid-induced glaucoma. In the results of qPCR, relative mRNA level of FKBP5 were incread, although there is no significance. It may be caused by the insufficient culture time. In previous report, FKBP5 was significantly upregulated in the DEX treated mice after 5 weeks DEX treatment [33]. In clinic, not every patient using corticosteroid eye drops will experience symptoms of increased Intraocular pressure, and it does not appear immediately upon use, corticosteroid glaucoma often appears in patients with long-term use [3]. So we suppose that there are some difference in the treatment between human TM cells and mice/human beings, since there are a series of feedback systems and related immune mechanisms in mice/human beings that work together to influence the final intraocular pressure and expression of FKBP5. The immune infiltration analysis revealed a significant decrease in macrophage M0 levels in the corticosteroid-induced sample, whereas macrophage M1 and M2 levels increased. Biological effects, particularly immediate responses involving the release of chemotactic and vasoactive agents like cytokines interleukin-1a, interleukin-1b, and tumor necrosis factor-a, may hold greater significance. These factors contribute to gelatinase release, macrophage recruitment, and various activities that influence aqueous outflow both directly and indirectly [5,34]. In our PPI network analysis, TSC22D3 was linked to FKBP5, leading to increased mRNA levels of TSC22D3. TSC22D3 exhibited a strong association with macrophage M2. Exogenous glucocorticoids enhance the expression of TSC22D3, a transcriptional suppressor with potent immunosuppressive properties [35]. Macrophages differentiate into subtypes, such as M1 and M2, based on environmental changes in various tissues. M1 macrophages initiate proinflammatory responses by producing cytokines such as interleukin 1 (IL-1), interleukin 12 (IL-12), tumor necrosis factor-α (TNF-α), chemokines (CXCL9–11, CCL2–5), reactive oxygen species (ROS), and nitric oxide (NO), facilitating pathogen elimination. M2 macrophages play a crucial role in anti-inflammatory responses and tissue regeneration. More specifically, M2 macrophages can polarize toward several subpopulations (M2a, b, and c), among these subpopulations, M2c macrophages polarize upon treatment with IL-10 or with stress-induced glucocorticoid. M2c macrophages secrete interleukin 10 (IL-10) and transforming growth factor-β (TGF-β), contributing to immune suppression and tissue remodeling [36,37]. Glucocorticoids can alter TSC22D3 gene expression, influencing macrophage activity and potentially affecting trabecular meshwork microstructure, thereby causing ocular hypertension. Glucocorticoid-induced TSC22D3 mediates immunosuppression was also reported in Yang’s research [35], suppoting our results. However, we found only one database, GSE37474, which set TM and corneoscleral tissue as experimental group, the result may lack in generalizability. No studies have examined the relationship between TSC22D3 and glucocorticoid-induced elevated intraocular pressure (IOP). Targeting TSC22D3 to suppress its overexpression in glucocorticoid treatments for ocular diseases may help prevent increased IOP. There will be some anti-inflammation actions after glucocorticoids treatment. It was reported that FKBP5 was involved in the negative feedback loops to regulate the action of glucocorticoid receptors [38]. TSC22D3 was implicated in the anti-inflammatory and anti-proliferative activities of glucocorticoids [38]. However, selective laser trabeculoplasty (SLT) is one kind of surgery to control the development of glaucoma. It was reported to demonstrated an increase in proinflammatory cytokine expression post SLT [39]. Thus, we supposed that an overly strong anti-inflammatory response may lead to IOP. It may need more experiment, such as gene knockout experiment to further confirm the related mechanism.

## Conclusion

In summary, through multiple dataset analyses, we identified genetic alterations in trabecular meshwork tissue following corticosteroid use, elucidated the biological functions involved, and identified the module most associated with trabecular meshwork changes using WGCNA analysis in corticosteroid-induced samples. We identified two key genes, FKBP5 and TSC22D3, within the module and conducted expression and ROC analyses to assess their monitoring efficacy. Our findings indicate a strong association between TSC22D3 and macrophage M2, highlighting the significant role of macrophages in the aqueous humor outflow process. We introduced a novel hypothetical mechanism for corticosteroid-induced ocular hypertension, providing significant insights into preventing glucocorticoid-induced glaucoma.

## Supporting information

S1 File S1-S4_Fig. docx The correction results of combining datasets from different platforms. (DOCX)
