# Supplementary material for: Identification and verification of hub biomarkers and immune-related pathways participating in the trabecular meshwork after using corticosteroid
Source: PLoS One. 2025 Sep 25;20(9):e0331281. doi: 10.1371/journal.pone.0331281 (PMC12463275; doi:10.1371/journal.pone.0331281)
Supplement: S1 File — (DOCX) [file pone.0331281.s001.docx]

S1-S4 Figure


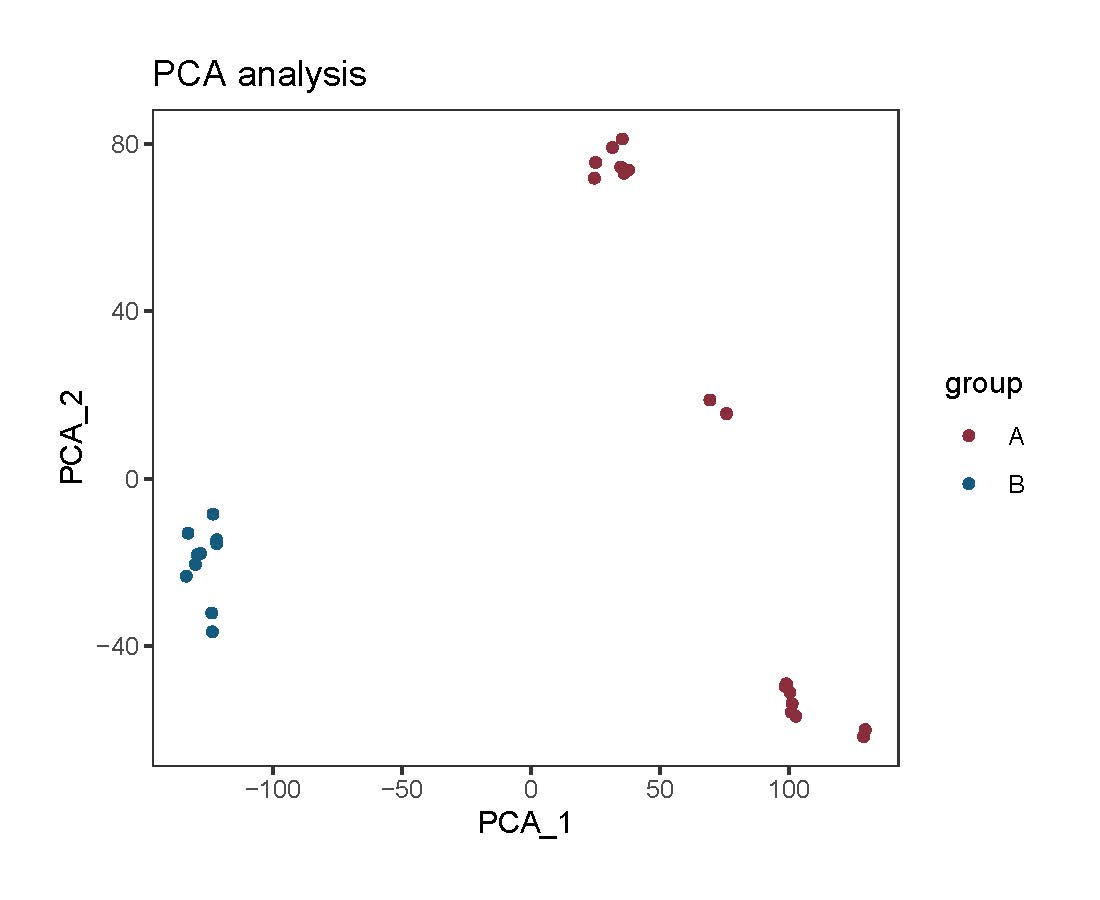


Fig. S1 PCA analysis of GSE374374, GSE124114 data for DEGs before correction. A: GSE124114, B: GSE374374.


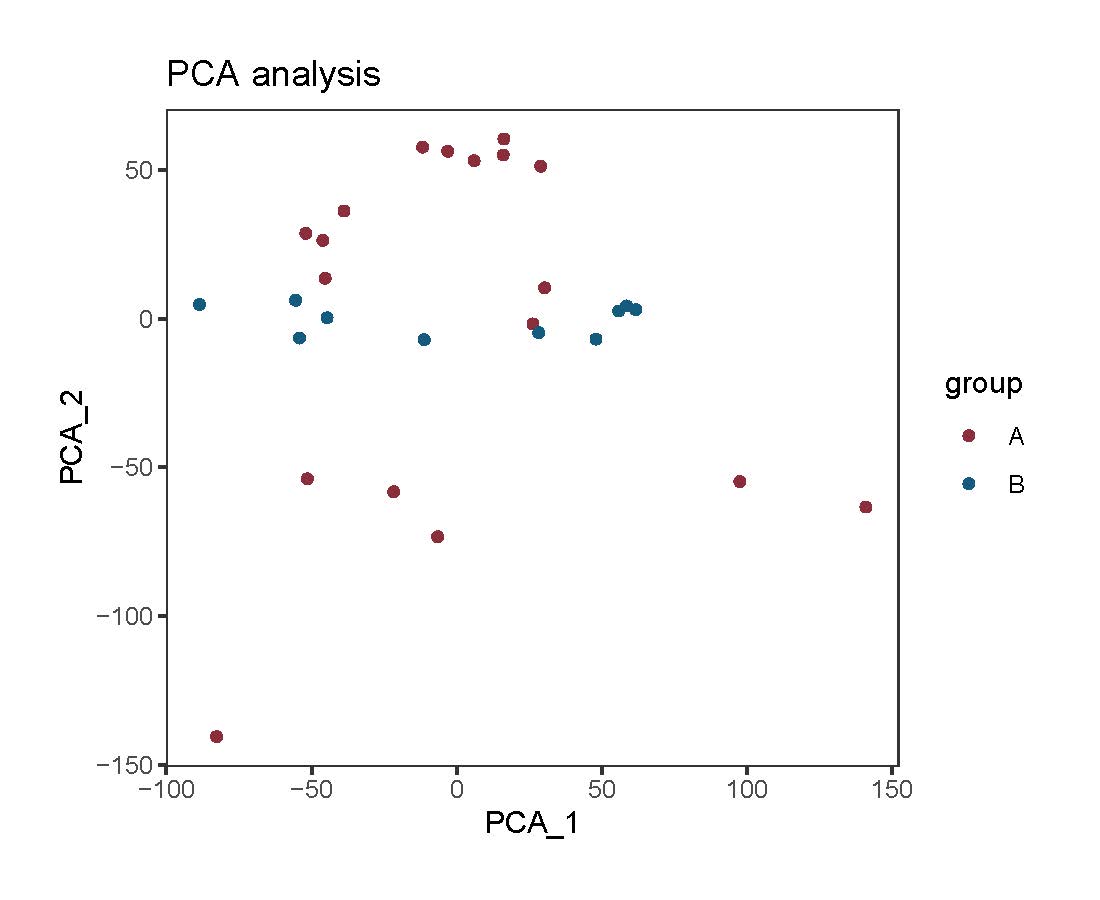


Fig. S2 PCA analysis of GSE374374, GSE124114 data for DEGs after correction. A: GSE124114, B: GSE374374.


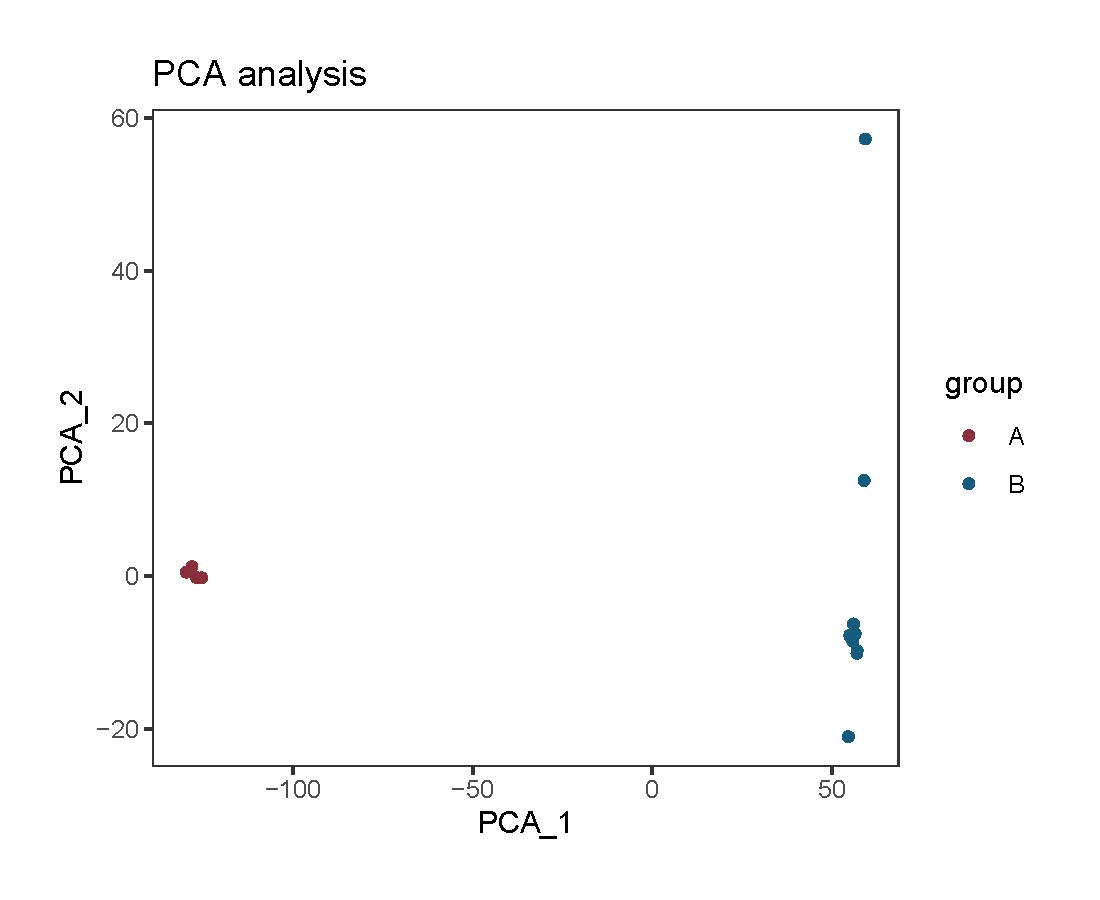


Fig. S3 PCA analysis of GSE6298, GSE665240 verify before correction. A: GSE665240, B: GSE6298.


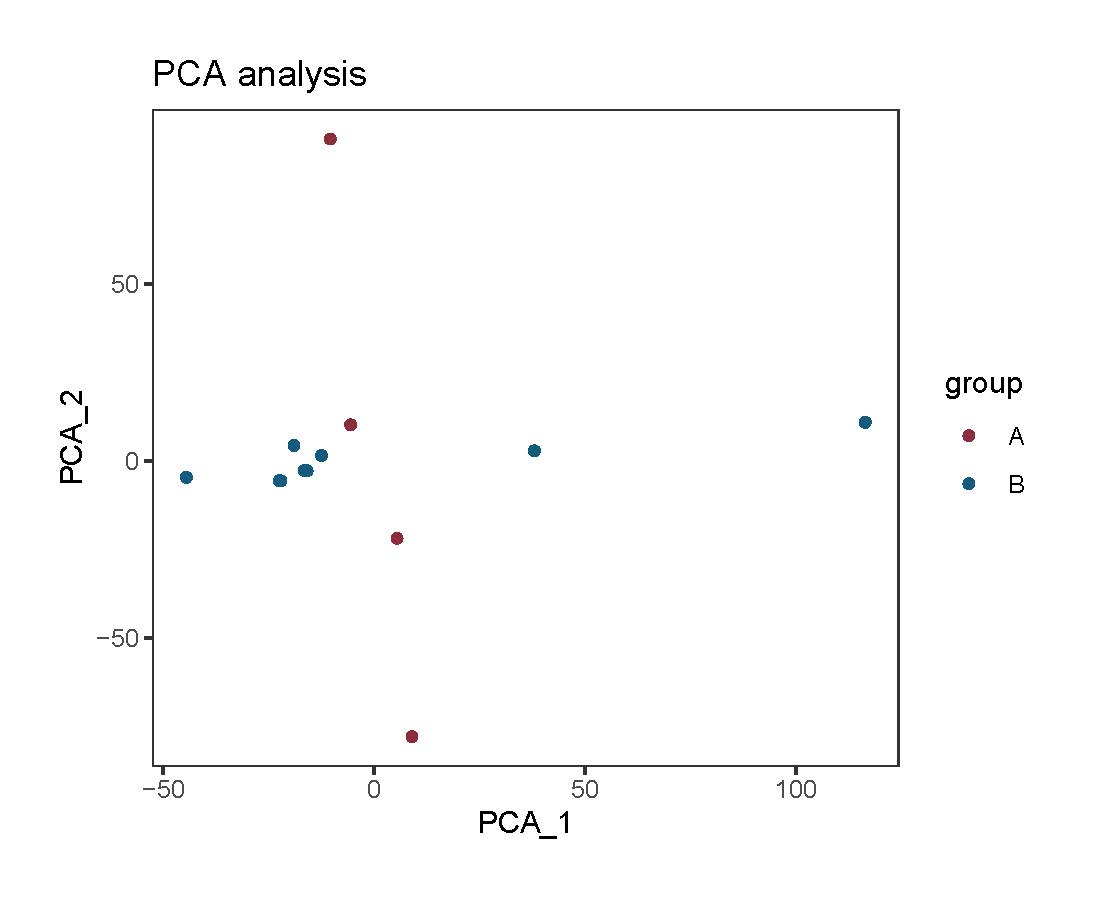


Fig. S4 PCA analysis of GSE6298, GSE665240 verify after correction. A: GSE665240, B: GSE6298.
